# Plants in Sleeping Rooms

**Published:** 1875-02

**Authors:** 


### PLANTS IN SLEEPING ROOMS.

Professor Kedzie, of the Michigan Agricultural College, has been conducting some experiments with a view to ascertain whether it is healthful or injurious to sleep in a room with growing plants.

Carbonic acid air or gas is the element which gives a small close chamber the dead heavy smell so perceptible when entering it of a morning from the fresh out-door air. If there is a large quantity of that kind of air in a room the sleeper will die in a few hours.

Charcoal gives out a large quantity of carbonic acid air—so much, indeed, that if a pan full is set on fire in a small room, and the doors, and windows, and other openings are closed, it will give out so much of that deadly air that any one breathing it would cease to live in a short time.

The air from a closed greenhouse was found to have nearly four parts of carbonic acid air in ten thousand parts of the whole, while there were a little over four parts of this gas in the pure country air outside.

Another experiment showed that the night air contained more carbonic acid air than the day air. The Professor's argument runs thus in his own words: "Now, if a room containing six thousand plants contains less carbonic acid than any sleeping room on this continent, we may safely conclude that a few plants in a chamber will not injure the sleepers." Any man who follows out this line of argument, by sleeping in that greenhouse a week, won't sleep much longer anywhere. Our own common sense teaches us that the best air to sleep in, other things being equal, is the out door air. It may contain more or less carbonic acid than a room containing growing plants, but for all that it is a purer air for the lungs—in fact, the out door air is the natural food of the lungs, and they are the wisest who get the most of it. We don't want to breathe air which simply does not injure, but that which we know to be most healthful.

